# Boosting the Charge Output of Enclosed Liquid‐Based Nanogenerators by Electrowetting‐Assisted Charge Injection Approach

**DOI:** 10.1002/advs.202506517

**Published:** 2025-07-29

**Authors:** Ye Zhao, Leiyang Wang, Guo Li, Chenlu Rao, Yuqi Pan, Borong Chen, Haihong Xu, Frieder Mugele, Hao Wu

**Affiliations:** ^1^ School of Physics and Optoelectronics South China University of Technology Guangzhou 510641 China; ^2^ Physics of Complex Fluids, Faculty of Science and Technology, MESA+ Institute for Nanotechnology University of Twente P.O. Box 217 Enschede 7500 AE The Netherlands; ^3^ State Key Laboratory of Advanced Papermaking & Paper‐based Materials South China University of Technology Guangzhou 510641 China

**Keywords:** electrowetting, energy harvesting, nanogenerator, solid‐liquid interface, surface charges

## Abstract

Liquid‐based nanogenerators (L‐NGs) have emerged as a promising solution for clean energy, appreciated for their minimal friction and effective contact at solid‐liquid interfaces. Enclosed L‐NGs, in particular, offer the benefits of enhanced durability and versatility. However, a key issue with enclosed L‐NGs is the low charge density resulting from triboelectrification at the liquid‐solid interface. In this study, this challenge is addressed by employing an electrowetting‐assisted charge injection (EWCI) approach to significantly enhance the charge output of the enclosed nanogenerator, which this study refers to as the EW‐NG. After EWCI treatment, the charge density has been enhanced by approximately ninefold, achieving a volumetric output charge density of 19.1 mC m^−3^, surpassing previous reports. The EWCI also ensures stable charge retention, contributing to the device's exceptional robustness, as evidenced by no significant degradation during intermittent testing over six months. Moreover, the high flexibility of the water within the device allows for operation in various modes and the generation of power from diverse mechanical energy sources. The EW‐NG has been successfully demonstrated to power an LCD screen with a size of 10 inches. This adaptability highlights the device's significant potential for applications in energy harvesting and self‐powered electronic systems in the field of the Internet of Things.

## Graphical abstract

The study introduces an electrowetting‐assisted charge injection (EWCI) approach to significantly enhance the charge density of enclosed liquid‐based nanogenerators (EW‐NGs). By leveraging EWCI, the volumetric output charge density reaches 19.1 mC m⁻^3^, a ninefold improvement over the conventional methods. Successfully powering a 10‐inch LCD screen, the EW‐NG highlights promising applications in IoT energy harvesting and self‐powered systems.

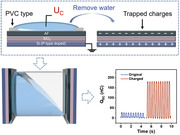

## 1 Introduction

In recent years, extensive research has concentrated on harnessing environmental energy to convert it into electricity, aiming to tackle the challenge of providing a distributed energy supply for electronic devices within the Internet of Things (IoT) ecosystem.^[^ ^1^, ^2^, ^3^, ^4^ ^]^ Liquid‐based nanogenerators (L‐NGs) represent an innovative approach for capturing clean and sustainable energy, generating electricity through interactions between liquids and solid materials or between different fluidic media.^[^ ^5^ ^]^ The liquid's dynamic properties ensure intimate, stable, and gentle interface contact, which allows L‐NGs to sustain an extended triboelectric effect and improve wear resistance.^[^ ^6^ ^]^ Moreover, the liquid medium, due to its inherent fluidity and adaptability to diverse materials and structures, provides a larger effective contact area.^[^ ^7^ ^]^ This characteristic enhances charge transfer and energy conversion efficiency, demonstrating its significant potential in applications such as blue energy, environmental monitoring, and biomedical sensors.^[^ ^8^ ^]^

Due to their low friction and high efficiency characteristics, L‐NGs have attracted growing interest. By leveraging solid‐liquid contact electrification and electrostatic induction, numerous L‐NGs with different materials and designs have been developed for capturing energy from water sources such as raindrops, ocean waves, and flowing water.^[^ ^9^ ^]^ In 2014, Lin et al. introduced a method for collecting kinetic energy of water drop falling, employing sequential contact electrification and electrostatic induction to enable dynamic interactions with solid surfaces.^[^ ^10^ ^]^ In 2020, Xu et al. proposed a transistor‐inspired device structure that markedly boosted the output voltage to hundreds of volts, offering new insights into energy harvesting from water droplets.^[^ ^11^ ^]^ In the same year, Wu et al. derived a scaling law for energy harvesting from water droplets, finding that optimum efficiency is achieved by matching the timescales of the external electrical energy harvesting circuit and the hydrodynamic spreading process.^[^ ^12^ ^]^ Subsequently, further studies have concentrated on enhancing the performance of L‐NGs by optimizing materials and refining structural designs.^[^ ^13^, ^14^ ^]^ Despite their achievements, L‐NGs systems with open‐ended structures, which rely on continuous external liquid supply and direct integration of functional materials with the ambient environment, face limitations in their applicability to various energy harvesting scenarios and exhibit lower durability.^[^ ^15^ ^]^

Compared to open‐air power generation systems, fully enclosed L‐NGs offer enhanced durability and strong resistance to environmental factors such as moisture and surface contamination, benefiting from their inherently sealed structure. In 2015, Jeon et al. developed the first enclosed L‐NG, which intentionally utilized liquid as the charge‐driving source, rather than relying directly on natural water source. These enclosed L‐NGs featured two opposing hydrophobic surfaces that sealed the cross‐sections at both ends of a tube, with the liquid occupying part of the internal space within this enclosed structure. The devices could capture ambient mechanical vibration energy by leveraging internal hydrodynamic oscillation through solid‐liquid contact electrification.^[^ ^16^ ^]^ Building upon this structure, subsequent researchers developed micro‐nano structures and interfaces with low surface energy to enhance the electrical output. For instance, in 2019, Liu et al. prepared the superhydrophobic surface by adhering polyvinylidene difluoride nanospheres to the microporous surface of a double‐faced adhesive tape, which could realize the voltage output of 30 V.^[^ ^17^ ^]^ Moreover, in 2022, Zhang et al. fabricated an enclosed L‐NG using molecular self‐assembly and the introduction of a dielectric silica layer, which exhibited outstanding electronegativity. The peak power density achieved was 43.0 mW m^−2^.^[^ ^18^ ^]^ However, currently, enclosed L‐NGs primarily rely on triboelectrification between liquid‐solid materials. The inherently unstable and low density of triboelectric charge at liquid‐solid interfaces severely limits the electric output of the device. Therefore, exploring approaches to enhance the density and stability of charges at liquid–solid interfaces is essential for achieving high‐performance L‐NGs for practical applications.

Here, we utilize the electrowetting‐assisted charge injection (EWCI) technology to enhance both the density and stability of the surface charge in the enclosed L‐NGs. After EWCI treatment, the nanogenerator, measuring 20 mm in diameter and 30 mm in length, achieves maximum outputs of 184.9 nC in charge, 101.5 V in voltage, 8.5 µA in current, and 0.102 mW in power, corresponding to a surface charge density of 0.59 mC m^−2^, and a peak power density of 10.87 W m^−3^. In particular, the volumetric charge density reaches 19.1 mC m^−3^, making it the highest among this type of energy harvesting device. To verify the stability of the surface charge, we subjected the electrowetting‐assisted nanogenerator (EW‐NG) to room conditions and tested it after six‐months. We found that the device maintained its performance, demonstrating its reliability. The flexibility of water allows the EW‐NG to operate in various modes, covering a broad spectrum of environmental mechanical activities. This adaptability indicates that the EW‐NG can be integrated into a wide range of applications as a self‐powered device, capable of harnessing energy from diverse sources. Utilizing the EW‐NG, we have successfully powered several LEDs and a 10‐inch LCD screen, showcasing its practical utility.

## 2 Results and Discussions

**Figure 1:**
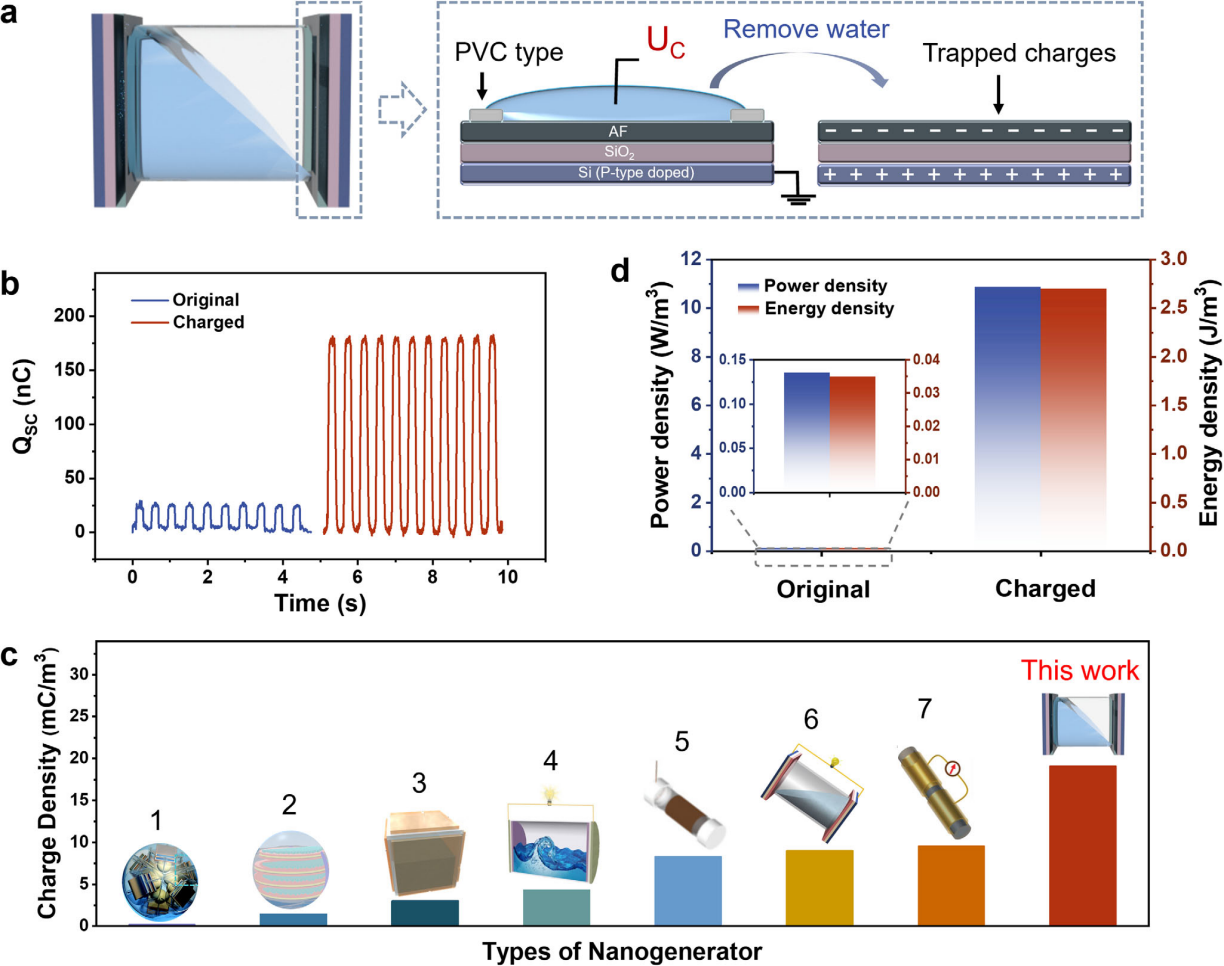
a) Schematic diagram of the fabricated EW‐NG and the EWCI process. The P‐type doped silica substrate served as the bottom electrode. The composite dielectric layer consisted of 1000 nm thick SiO_2_ (bottom layer) and 120 nm AF films (top layer). The edges are sealed with white PVC tape. b) Comparison of the short‐circuit transferred charges (*Q*_SC_) of the enclosed L‐NGs using original AF and charged AF as electric materials. c) Comparison of the volume density of the transferred charge of this work to other reports. (Reference information: No.1:^[^ ^26^ ^]^ Reproduced with permission. Copyright 2020, Royal Society of Chemistry. No.2:^[^ ^27^ ^]^ Reproduced with permission. Copyright 2021, American Chemical Society. No.3:^[^ ^16^ ^]^ Reproduced with permission. Copyright 2015, Elsevier. No.4:^[^ ^28^ ^]^ Reproduced with permission. Copyright 2020, Elsevier. No.5:^[^ ^29^ ^]^ Reproduced with permission. Copyright 2022, John Wiley and Sons. No.6:^[^ ^18^ ^]^ Reproduced with permission. Copyright 2022, Elsevier. No.7:^[^ ^30^ ^]^ Reproduced with permission. Copyright 2021, John Wiley and Sons. d) Comparison of power density and energy density of NGs of AF original and AF charged as electric materials. (the tube length is 30 mm, the diameter is 20 mm, volume fraction of liquid of 40%, swing angle is 60°, frequency 2 Hz, 100 mm NaCl solution, and load resistance 10 MΩ, movement type: seesaw).

We create two amorphous fluoropolymer (AF)/ SiO_2_/ doped Si substrates with electrowetting‐induced trapped charges, then, fabricate a fully enclosed liquid‐based nanogenerator (EW‐NG) by using a hydrophobic fluorinated ethylene propylene (FEP) tube and positioning the two substrates at either end of the tube. **Figure** **1**a demonstrates the schematic diagram of the fabricated EW‐NG and EWCI technique for generating surface charges.^[^ ^19^ ^]^ Figure S1 (Supporting Information) provides a visual representation of the EWCI process, where polyvinyl chloride (PVC) tape masks are used to delineate the specific surface area for charging, which is typically ≈4 cm^2^. The EWCI process begins by depositing a large water droplet, several milliliters in volume, onto the surface of an AF film. Then, a charging voltage (U_C_) of up to −800 V (relative to the grounded bottom electrode) is applied across the dielectric layers through the water droplet for a duration of 10 min. Afterward, the charging process is completed by removing both the voltage and the water droplet. The U_C_ must be sufficient to inject charge into the AF film, but it should not be set too high, as excessive voltage could cause the dielectric films to break down.^[^ ^20^ ^]^ The leakage current under varying U_C_ levels is depicted in Figure S2 (Supporting Information).

The EW‐NG could achieve a reciprocating mechanical oscillation by external mechanical trigger. Using a linear motor equipped with a swinging frame to drive the EW‐NG with a constant frequency and swing angle, we evaluate the electric output of the EW‐NG, including the transferred charge (*Q*_SC_), open‐circuit voltage (*U*_OC_), and short‐circuit current (*I*_SC_). Figure 1b and Figure S3 (Supporting Information) compare the electric output of the EW‐NG and a control sample without EWCI treatment. The EW‐NG's maximal *Q*_SC_, *U*_OC_, and *I*_SC_ values reach 184.9 nC, 101 V, and 8.5 µA, respectively, which were all approximately 9 times higher than those obtained from the control samples. Thanks to our EWCI process that enables a high surface charge density, the volumetric charge density reaches 19.1 mC m^−3^, which is higher than existing reports on wave energy harvesting (Figure 1c; Table S1, Supporting Information). Except for the charge, voltage, and current output, we also compare the volume power density and energy density (Figure 1d). Using a load resistance of 10 MΩ, the power and energy output density under a seesaw‐driven mode reaches 10.87 W m^−3^ and 2.7 J m^−3^, respectively, which is ≈80 times of the controls. This result indicates that the EW‐NG exhibited stronger ability to enhance the electric output of the enclosed L‐NGs.

**Figure 2:**
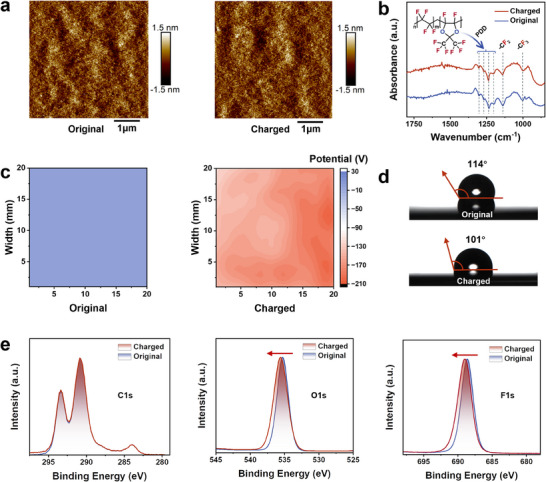
a) AFM images showing the surface topographies of AF films before and after EWCI process. b) Fourier transform infrared (FTIR) spectroscopy of AF films before and after the EWCI. c) The surface potential of AF films before and after EWCI process. d) Contact angles of the water on the surface of AF films before and after EWCI process. e) C1s, F1s, and O1s XPS spectra of AF films before and after EWCI process.

To assess the influence of the EWCI process on the surfaces of AF films, we performed a series of surface characteristic analyses. The surface topography was observed by atomic force microscopy (AFM) and scanning electron microscopy (SEM), and the results are depicted in **Figures** **2**a and S4 (Supporting Information). These observations indicate the EWCI process does not alter the topography of the AF film surface. Additionally, Fourier transform infrared (FTIR) spectroscopy was employed to determine if the EWCI process triggers electrochemical reactions within the AF films, with results presented in Figure S5 (Supporting Information). The wavenumber range of 1800–850 cm^−1^ was scrutinized for detailed analysis (Figure 2b). The absorption at 1140 cm^−1^ arises from the symmetric and asymmetric stretching vibrations of the CF_2_ monomer. The AF spectrum exhibits a strong peak at ≈990 cm^−1^, characteristic of CF_3_ vibrations. The remaining peaks (1120, 1240, 1270, and 1305 cm⁻¹) were observed exclusively in the AF material and can be attributed to the fluorine‐containing dioxole ring (PDD unit).^[^ ^21^, ^22^ ^]^ The absence of any wavenumber shift in the FTIR data suggests that no new functional groups are formed following the EWCI process. Furthermore, scanning electron microscopy with energy‐dispersive X‐ray analysis (SEM‐EDX) was used to analyze the morphological characterization and elemental mapping of the AF films before and after the EWCI process, as shown in Figure S6 (Supporting Information). The SEM‐EDX findings demonstrate that neither the morphology nor the chemical properties of the AF films were altered during or after the charging process.

To substantiate the charge injection and to profile the surface charge, the surface potential distribution across the AF films was measured before and after the EWCI treatment using a Kelvin probe. The surface potential of the AF film was found to be near zero before the EWCI process, as illustrated in Figure 2c. In contrast, after undergoing EWCI, the surface potential of the polymer films was significantly enhanced to −200 V. This reduction in surface potential is indicative of a successful charge injection, thereby validating the effectiveness of the EWCI approach. Although the chemical composition of the film is unaffected by the process, there are noticeable shifts in its physical surface properties. Notably, as shown in Figures 2d and S7 (Supporting Information), the water contact angle has decreased, a reduction that we attribute to the induced negative surface charges. These surface charges create an electrostatic potential, which in turn causes the contact angle to decrease as a result of the electrowetting effect. Similar observations have been reported in earlier research. Moreover, X‐ray photoelectron spectroscopy (XPS) was used to analyze the AF films before and after EWCI (Figure 2e; S8, Supporting Information). XPS spectra show main peaks of C1s, F1s, and O1s. After negative charge injection, the F1s and O1s peaks shift by 0.5–1.0 eV toward higher binding energy, while the C1s peak remains stable as a calibration reference. The observed energy shift stems from space charge buildup in the dielectric layer, creating a sustained internal electric field that causes differential charging during XPS analysis.^[^ ^23^ ^]^ Crucially, no new peaks or peak splitting are observed, confirming that the charging process does not alter the material's chemical composition.

**Figure 3:**
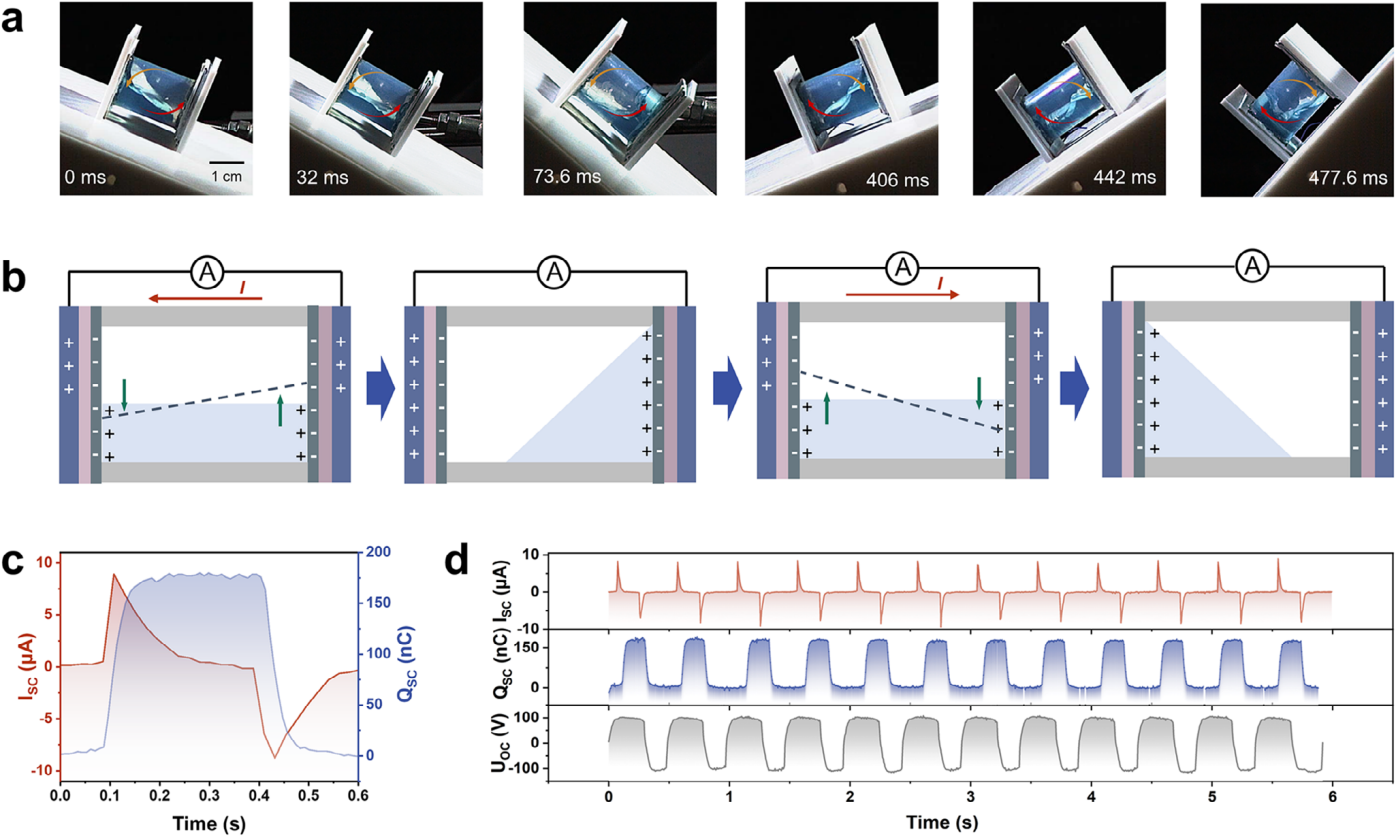
a) Photographs showing the movement process of the liquids in EW‐NG. b) Electricity generation mechanism of EW‐NG. c) The measured short‐circuit current (*I*_SC_) and charge (*Q*_SC_) during a complete reciprocating oscillation cycle. d) *I*_SC_, *U*_OC_, and *Q*_SC_ of an individual EW‐NG working in seesaw motion mode at a frequency of 2 Hz.

After obtaining substrates with charged surfaces, we fabricate a fully encapsulated nanogenerator (EW‐NG) by equiped the charged surfaces with a hydrophobic FEP tube and filling it with water, which transforms mechanical vibrations into electrical energy. As shown in Figure S9 (Supporting Information), the contact angles of the water on the surface of FEP is 108°. The FEP tube's hydrophobicity is deliberately chosen to reduce water adhesion, which is crucial for the reliable cycling of the solid‐liquid interface through contact and separation, and further ensures the electrical output of the nanogenerator. The underlying mechanism involves the motion‐induced displacement of water within the device, causing it to contact and then separate from the charged AF surfaces. To understand the movement process of liquids more intuitively, a high‐speed camera was employed to capture liquids movement trajectory, which clearly shows that the contact and separation process modulates the solid‐liquid interface under the external mechanical motion (**Figure** **3**a). This contact and separation process modulates the electric double layer (EDL) at the liquid‐solid interface, which in turn generates an electric potential difference and initiates charge transfer across the electrodes beneath the AF surface.^[^ ^24^ ^]^ The details of this mechanism are depicted in Figure 3b.

The generated current *I*_SC_ can be expressed by the equation:

(1) $$I_{SC}=\frac{dq}{dt}=\sigma_{e}\frac{dS}{dt}$$

where *q* is the transferred charge, *t* is the time, σ_e_ is the effective surface charge density, *S* is the water/AF contact area. Equation 1 indicates that, with σ_e_ held constant, the current is primarily influenced by the rate of interfacial area change at the water/AF interface. The current reaches its peak when the interface experiences the most rapid change. Figure 3c illustrates the current and charge output during a complete reciprocating oscillation cycle. Additionally, we employed multi‐physics finite element simulation (COMSOL software) to simulate the potential distribution of the system (Figure S10, Supporting Information). The simulation results confirm the validity of the proposed working principle. To evaluate the electric generation capacity of the EW‐NG, we test the output of short‐circuit current (*I_SC_*), open‐circuit voltage (*U_OC_*), and charge (*Q_SC_*), as shown in Figure 3d. The current, voltage, and charge output reach 8.5 µA, 101 V, and 185 nC, respectively.

**Figure 4:**
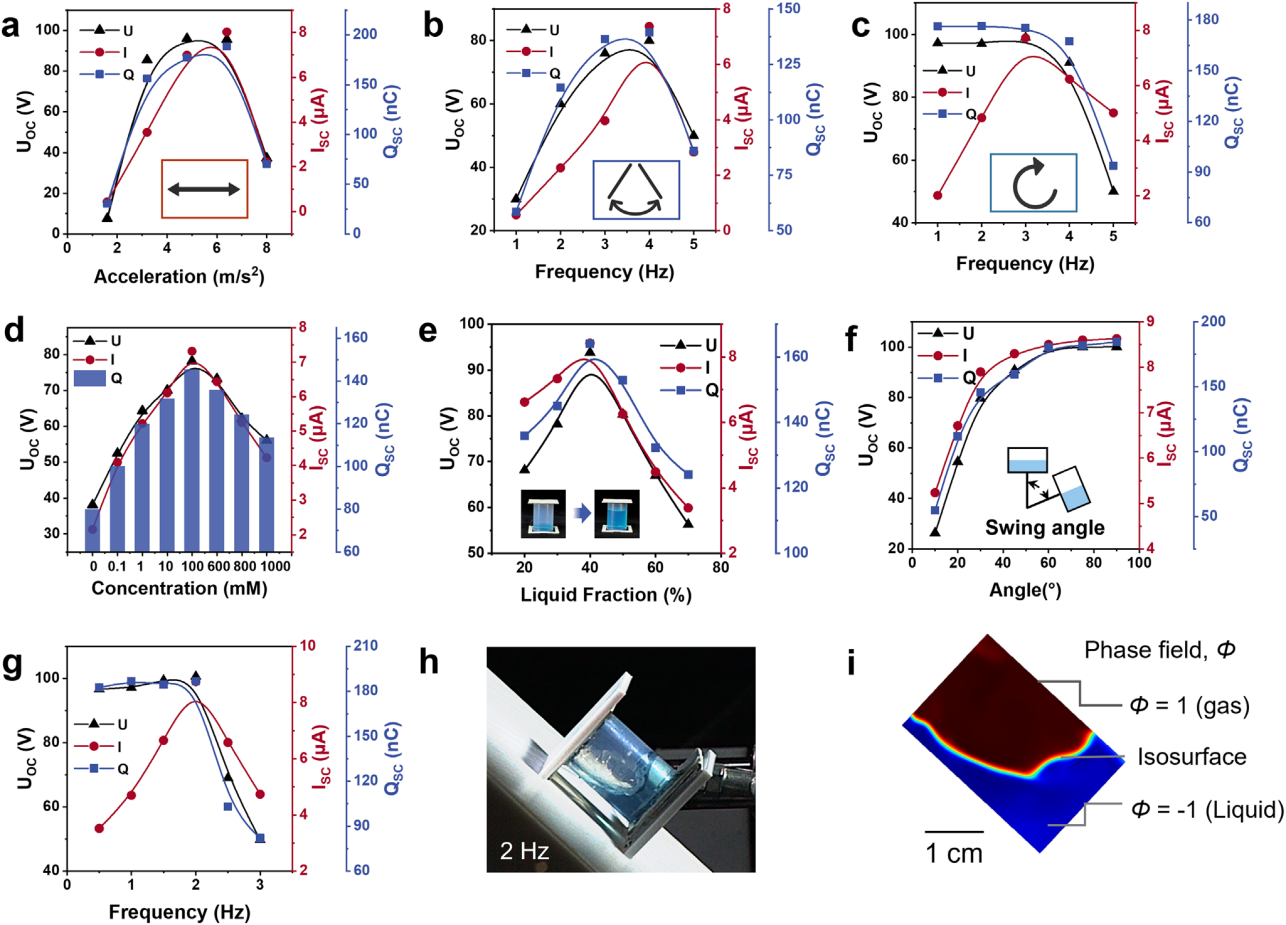
a–c) The maximum *Q*_SC_, *I*_SC_, and *U*_OC_ depending on the frequency of EW‐NG operated in horizontal linear mode, swing modes, and rotation. d) The *Q*_SC_, *U*_OC_, and *I*_SC_ of EW‐NG depending on NaCl concentration of the water solution (keep tube diameter of 2 cm, FEP tube length of 3 cm, the volume fraction of liquid is 30%, swing angle 30°, 2 Hz) e) EW‐NG outputs depending on a volume fraction of liquid (keep Swing angle 60°, Frequency 2 Hz, 100 mm NaCl solution, tube diameter:20 mm, length:30 mm). f) Swing angle (keep volume fraction of liquid of 40%, Frequency 2 Hz, 100 mm NaCl solution), seesaw mode. g) *Q*_SC_, *U*_OC_, and *I*_SC_ of EW‐NG under various oscillation frequencies from 0.5 to 3 Hz. h) High‐speed photo taken by EW‐NG in seesaw fluid motion state at 2 Hz. i) The corresponding 2 Hz frequency COMSOL simulation.

The EW‐NG's adaptability in water allows it to function effectively across different modes, as demonstrated by the electric outputs shown in **Figure** **4**a–c for horizontal linear, swing, and rotational operations. In the linear and swing modes, higher acceleration or frequency enhances water motion and the solid‐liquid contact area, boosting the charge, current, and voltage outputs. However, excessively fast water movement can lead to fluctuations that reduce the effective contact area and, consequently, reducing the output. In rotational mode, the device leverages gravity‐induced water motion to generate power even at low frequencies. Yet, beyond a rotation frequency of 3 Hz, centripetal force pushes the water toward the tube's outer side, diminishing the solid‐liquid contact area and output. Thus, the EW‐NG is optimized for low‐frequency energy harvesting. These modes align with a range of natural mechanical energies, such as ocean waves, arm swings, and wind‐driven rotations. The EW‐NG's ability to capture energy from these complex motions without additional mechanical interfaces is a significant advantage. This feature enables the device to be versatile, fitting a variety of scenarios and serving as a versatile solution for energy harvesting in diverse settings.

We then assess the variables that impact the performance of the EW‐NG, such as the solution's ionic strength, the quantity of solution, the excitation frequency, and the amplitude of the operational swing. Figures 4d and S11 (Supporting Information) depict the charge, voltage, and current outputs of the EW‐NG across a range of NaCl solution concentrations from 0 to 1000 mm. Initially, the electrical output rises with the concentration of NaCl, but it begins to decline once the concentration exceeds 100 mm. The initial rise in output is attributed to the increased availability of ions at higher concentrations, which enhances the formation of the electric double layer (EDL) at the water/solid interface. The dynamic formation and collapse of the EDL drive the induced charge transfer in the electrodes, leading to higher electrical outputs at lower concentrations. However, at very high concentrations, an excess of ions can lead to a screening effect at the interface. Here, the surplus cations neutralize the negative charges on the AF surface, which diminishes the effectiveness of the EDL and consequently reduces the electrical output. Next, we examine the impact of the solution's volume fraction. Figure 4e demonstrates that the electrical output of the device is directly proportional to the water volume fraction up to a certain threshold. The output increases as the water volume fraction rises, reaching a peak at 40%. Beyond this threshold, the output declines. This trend is explained by the fact that when the water volume is too low, it is insufficient to fully cover the surface of the charged AF film, which is necessary for optimal interaction. Conversely, when the water volume exceeds 40%, the excess water can cause the AF surface on one side to be covered before the water on the other side has fully detached. This premature coverage reduces the total change in the AF/water interface, which in turn leads to a decrease in the device's output. Additionally, the swing angle significantly influences the electrical output, as shown in Figure 4f. The output increases with the swing angle, peaking when the angle reaches 60°. At this optimal angle, the solid‐liquid interface achieves full contact and separation, maximizing the output. However, when the swing angle is below 60°, the water fails to fully contact and detach from the AF surface, resulting in a decreased electric output.

The impact of oscillation frequencies on the output was also examined, with results shown in Figure 4g and Figure S12 (Supporting Information). When the frequency increased from 0.5 to 2 Hz, the *Q*_SC_ and *U*_OC_ remained largely unchanged, while the *I*_SC_ increased linearly from 3.5 to 8.5 µA. The stability of the *U*_OC_ and *Q*_SC_ is due to the fact that the total charge transfer is determined by the change in the water/AF interfacial area and the charge density at the interface. As long as the total interfacial area and charge density remain constant, the total charge transfer, and thus the voltage output, remains unchanged. In contrast, the current output is influenced by the rate of change of the interfacial area; a higher frequency leads to a faster rate of change, which in turn increases the current output. However, when the frequency exceeded 2 Hz, we observed a sharp decrease in the output of voltage, charge, and current. This decline is due to the rapid shaking causing water fluctuation within the tube. At such high frequencies, the water on one side had not completely detached from the AF surface before the water on the other side began to make contact, reducing the total variation of the water/solid interface and, consequently, the output. To verify this, we used a high‐speed camera to monitor the hydrodynamics of the water at different frequencies, which confirmed our hypothesis (Figures 4h; S13a and Video S1, Supporting Information). Furthermore, we conducted multi‐physical simulations that yielded similar results, supporting our assumptions and experimental findings (Figure 4i; S13b, Supporting Information).

**Figure 5:**
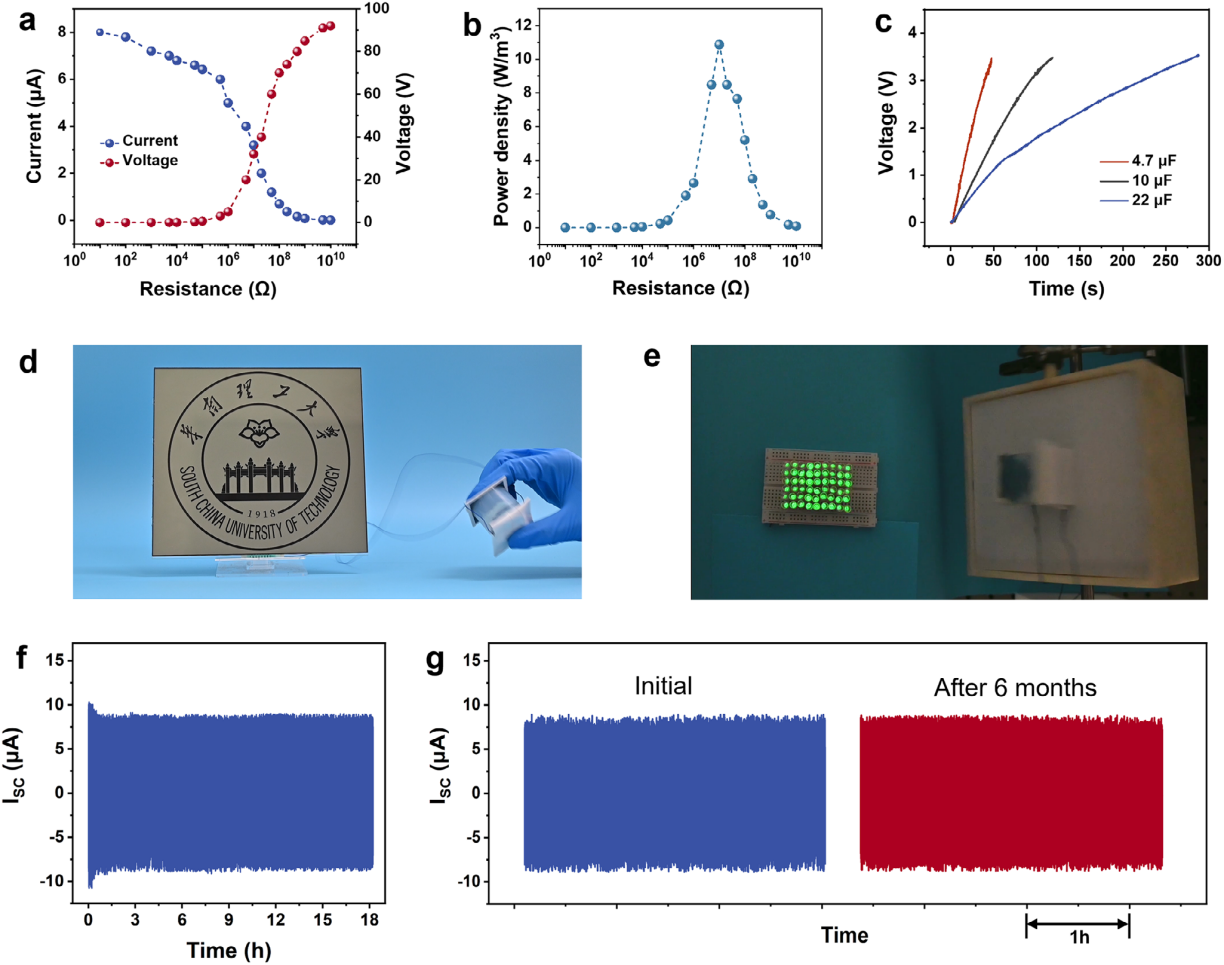
a) Output peak voltages and currents of an individual EW‐NG with different external load resistance. b) Peak power density of an individual EW‐NG at different external load resistances. c) An EW‐NG charged the different commercial capacitors (4.7, 10, and 22 µF). d,e) Photographs of LCD screen with a size of 10 inches and 56 commercial LEDs powered by an individual EW‐NG. f) *I*_SC_ of EW‐NG during 1.3 × 10^5^ cycles and g) Cycles of 3 h after six months without electrical output attenuation. (The EW‐NG's specification: Tube inner diameter of 20 mm, and tube length of 30 mm, volume fraction of liquid of 40 %, Frequency 2 Hz, 100 mm NaCl solution).

The current and voltage output of the EW‐NG depending on various load resistances was evaluated, as shown in **Figure** **5**a. The maximum instantaneous power output reaches 10.87 W m^−^^3^ with a load resistance of 10 MΩ (Figure 5b). To assess the practical performance of the EW‐NG, we conducted tests on its capacitance charging capabilities and showcased its potential as a power source for self‐powered electronic systems. Figure 5c presents the capacitor charging results. To charge capacitors of 4.7, 10, and 22 µF to 3.5 V, the EW‐NG requires operation times of 47.5, 115, and 287 s, respectively, under a frequency of 2 Hz. Potential applications include harnessing energy from ocean waves or the oscillations of marine vehicles with the EW‐NG, then storing this electricity in an energy storage device for powering ocean sensors.

Enhancing the size of the device can enhance the power output. As shown in Figure S14, the EW‐NG has higher current and charge output with a diameter of 34 mm and a length of 30 mm, which are nearly proportional to the pipe diameter and area, respectively. The effect of frequency on the maximum charge output is similar to the previously prepared smaller size. Furthermore, an individual EW‐NG could power an LCD screen with a size of 10 inches and light up 54 commercial LEDs easily (Figures 5d,e, and Video S2 and Figure S3 Supporting Information). For practical applications, the output power can be effectively scaled by connecting multiple EW‐NGs in parallel. The alternating current (AC) output generated can be converted to direct current (DC) using a bridge rectifier circuit, enabling energy storage in capacitors or batteries, as well as direct power supply to low‐power electronic devices. Thanks to the reliable surface charge generated by the EWCI approach, our EW‐NG device exhibits excellent stability. As shown in Figure 5f, the durability of EW‐NG was tested over an 18 h measurement period, during which the *I*_SC_ showed a slight decrease before leveling off to remain essentially constant. Furthermore, it has demonstrated a stable current output with minimal attenuation even after six months of intermittent testing (Figure 5g). This further confirms the stability of EWCI‐generated charges, consistent with previous findings.^[^ ^20^, ^25^ ^]^ In addition, the fully encapsulated design based on water‐solid interface demonstrates remarkable environmental stability, particularly under high‐humidity conditions, where conventional solid‐based devices typically suffer from performance degradation (Figure S15, Supporting Information). This sustained performance indicates good durability and reliability, making the EW‐NG suitable for practical applications. This has great potential for the development of self‐powered electronic systems in the field of the Ambient Internet of Things.

## 3 Conclusion

In summary, we developed a novel electricity generator with boosted charge output by EWCI approach. The EW‐NG addresses significant limitations of conventional nanogenerators, such as low power density, low and unstable surface charge density, and poor long‐term reliability. It exhibited an excellent volumetric output charge density of 19.1 mC m^−3^, surface charge density of 0.59 mC m^−2^, and peak power density of 10.87 W m^−3^. The highest among nanogenerators for low‐frequency energy harvesting to date. Moreover, the EW‐NG demonstrated excellent reliability over six months of testing. The flexibility of the water allows the EW‐NG to operate in multiple modes, including rotation, swing, seesaw, and horizontal linear modes. These modes encompass a wide range of environmental mechanical movements, suggesting that the EW‐NG can be integrated into diverse applications as a self‐powered device, harvesting energy from various sources. Benefitting from the high charge and power density, the application of the EW‐NG was demonstrated, showing potential for use in energy harvesting and self‐powered electronic systems in the field of the Ambient Internet of Things.

## 4 Experimental Section

### Materials

Teflon AF1600 (the Chemours Company, USA), Doped silicon wafers (P‐type, resistivity was less than 0.0015 Ω cm) with thickness of SiO_2_ layer (1000 nm) were purchased from Shenzhen Shun Sheng Electronics. Methylene Blue was purchased from Adamas‐beta. Sodium Chloride utilized in this study was purchased from Zhengzhou Feynman Biotechnology Co., Ltd (Zhengzhou, China; mall. shiyanjia.com).

### Characterization

EWCI technology involves high‐voltage power supply was American Stanford SRS DC High Voltage Power PS365. The surface states of Teflon AF1600 before and after EWCI process were studied by X‐ray photoelectron spectroscopy (XPS, Axis Supra+, Kratos). Atomic Force Microscope (AFM) images showing the surface topographies were characterized by Bruker Icon. The scanning electron microscopy (SEM) images and energy dispersive X‐Ray analysis (EDX) of samples were obtained using a Zeiss Gemini‐SEM 500. Fourier transform infrared (FTIR) spectroscopy of Teflon AF1600 films before and after the EWCI were characterized by Nicolet IS50 ‐ Nicolet Continuum. The surface potential of Teflon AF1600 before and after EWCI process were obtained using a Kelvin probe (APS04‐N_2_‐RH, KP technology). Film thickness of Teflon AF1600 were characterized by Bruker Dektak XT. The fluid motion state was captured by a color high‐speed camera Memrecam ACS‐3 M16. The solution in the tube was stained with methylene blue at a concentration of 10 ppm to better illustrate the fluid dynamics. Constant temperature and humidity control by KINGjo programmable constant temperature and humidity test chamber CK‐80T.

### The method of EWCI

A doped silicon (Si) wafer functions as the bottom electrode, while a thermally grown silicon dioxide (SiO_2_) layer serves as the dielectric layer. An AF film, with a thickness of 120±10 nm, was spin‐coated onto the SiO_2_ surface. Polyvinyl chloride (PVC) tape masks were used to delineate the surface area intended for charging, typically spanning an area of 4 cm^2^. A large droplet of water, amounting to several milliliters, was placed on the AF surface, covering the entire area including the PVC masks, to prevent the formation of a free and mobile air‐water‐solid three‐phase contact line. Following this, a charging voltage (U_C_) of up to −800 V (relative to the grounded bottom electrode) was applied across the dielectric layers through the water puddle for a duration of 10 min. Upon completion of the charging process, both the voltage and the water were removed. Throughout this procedure, the leakage current remains below 0.015 mA.

### Fabrication of the EW‐NG

The schematic of the design and fabrication process of the EW‐NG is shown in Figure 1a. The EW‐NG was assembled using Teflon AF1600 after EWCI process with silicon dioxide silicon wafer substrate as the solid generate electricity material and 100 mmol sodium chloride solution as the liquid generation electricity material. First, two pieces of AF‐SiO_2_‐silicon substrates were fixed on both sides of the FEP tube and sealed with glass glue to prevent water leakage. Then 20 mL of 100 mmol sodium chloride solution was filled into the sealed tube to form the EW‐NG.

### Measurement of the Electric Output

The device (Figure 1a) was driven by a motor with controllable frequency and rotation angle. The voltage, current, and transferred charge were all measured using a electrometer (Keithley 6514). The output energy can be given by

(2) $$E=R\int_{0}^{T}I{\left(t\right)}^{2}dt$$

where *E* is the output energy, *I(t)* is the output current, *R* is the external load resistance, *T* is the period.

All electrical performance experiments were conducted under standardized conditions (25 ± 0.5 °C, 50 ± 5% relative humidity) unless otherwise noted. COMSOL MULTIPHYSICS software was employed for potential distribution simulation.

## Conflict of Interest

A China patent application related to this research (CN117526760A) has been filed.

## Author Contributions

Y. Z. and L. W. contributed equally to this work. H. W. conceived the project. Y.Z., G.L., and H. W. designed the experiment. Y.Z., L.W., and Y.P. prepared the samples and performed the experiment. Y.Z., G.L., C.R., H.X., and F. M. analyzed the results. C.R. drew mechanism diagram. B.C. designed 3D printed brackets. Y.Z., F.M., and H. W. wrote and modified the manuscript.

## Supporting information



Supporting Information

Supplemental Data1

Supplemental Video1

Supplemental Video2

Supplemental Video3

## Data Availability

Data supporting the findings of this study can be obtained from the corresponding authors with reasonable requests.
