# Supplementary material for: Boosting the Charge Output of Enclosed Liquid‐Based Nanogenerators by Electrowetting‐Assisted Charge Injection Approach
Source: Adv Sci (Weinh). 2025 Jul 29;12(38):e06517. doi: 10.1002/advs.202506517 (PMC12520542; doi:10.1002/advs.202506517)
Supplement: Supplementary file 1 — Supporting Information [file ADVS-12-e06517-s004.docx]

**Supporting information**

**Boosting the Charge Output of Enclosed Liquid-based Nanogenerators by Electrowetting-assisted Charge Injection Approach**

*Ye Zhao*, *Leiyang Wang*, *Guo Li*, *Chenlu Rao*, *Yuqi Pan*, *Borong Chen*, *Haihong Xu*, *Frieder Mugele*, *Hao Wu**

Y. Zhao, L. Wang., G. Li., C. Rao, Y. Pan, B. Chen, H. Xu, H. Wu

School of Physics and Optoelectronics, South China University of Technology, Guangzhou 510641, China.

*Corresponding author. E-mail: [haowu@scut.edu.cn](mailto:haowu@scut.edu.cn)

F. Mugele

Physics of Complex Fluids, Faculty of Science and Technology, MESA+ Institute for Nanotechnology, University of Twente, P.O. Box 217, Enschede 7500 AE, The Netherlands.

Y. Zhao and L. Wang contributed equally to this work.


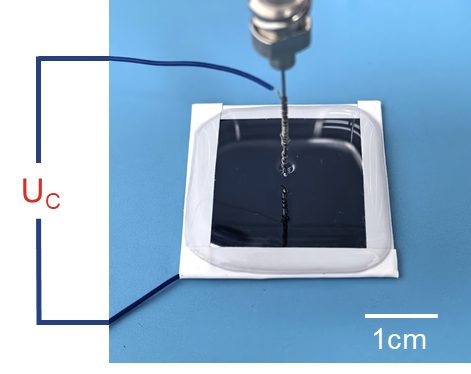


**Figure S1.** Picture of a sample being charged by using the EWCI method.

**Figure S2.** Leakage current during EWCI charging process. charging voltage: -800 V; Charge time: 10 minutes; charging liquid: DI water; Sample: 117 ± 10 nm Teflon AF1600 + 1000 nm SiO_2_.


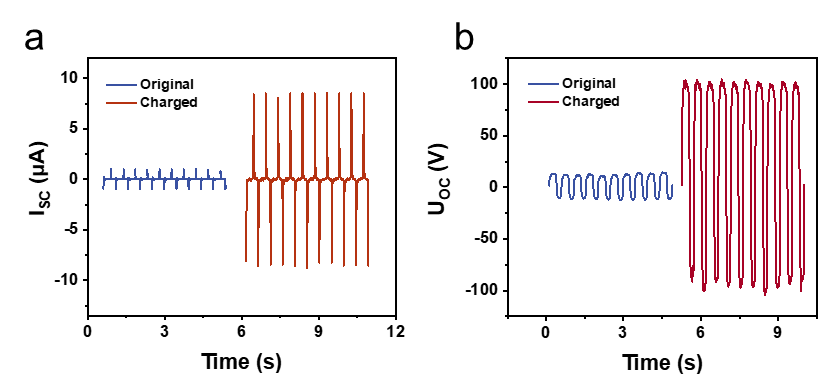


**Figure S3.** Comparison of I_SC_ and U_OC_ of EW-NG with original and charged AF films as electric materials.


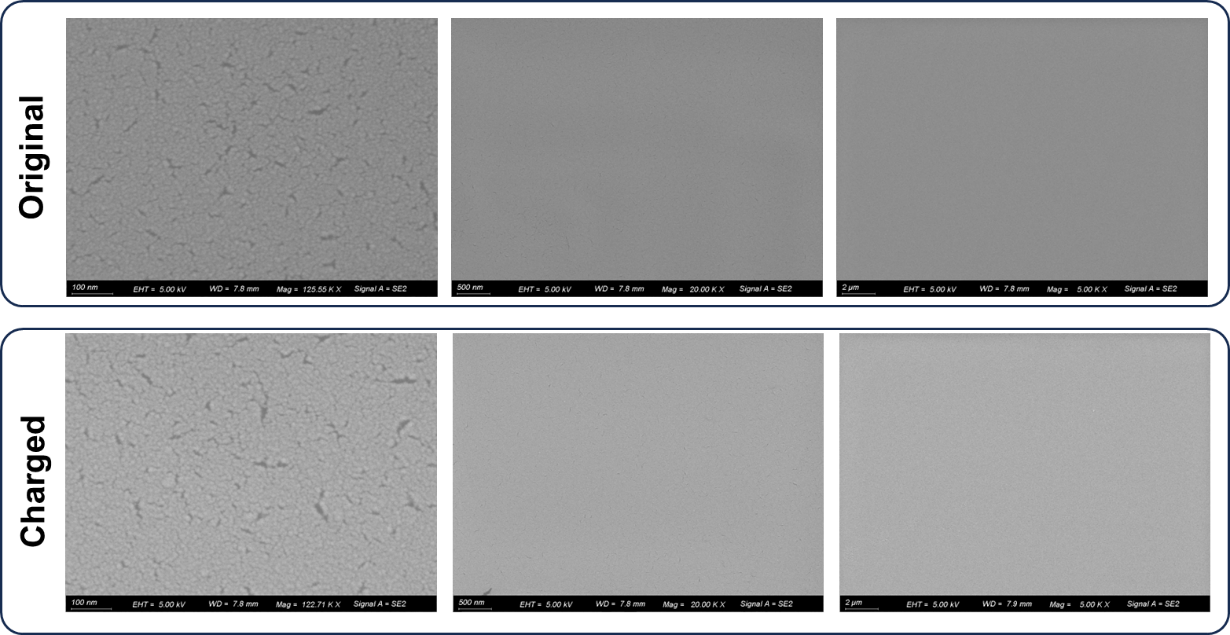


**Figure S4.** SEM images of Teflon AF1600 before and after EWCI process. The scales are 100 nm, 500 nm and 2 μm.

**Figure S5.** FTIR-GRA of Teflon AF1600 before and after EWCI charged process.


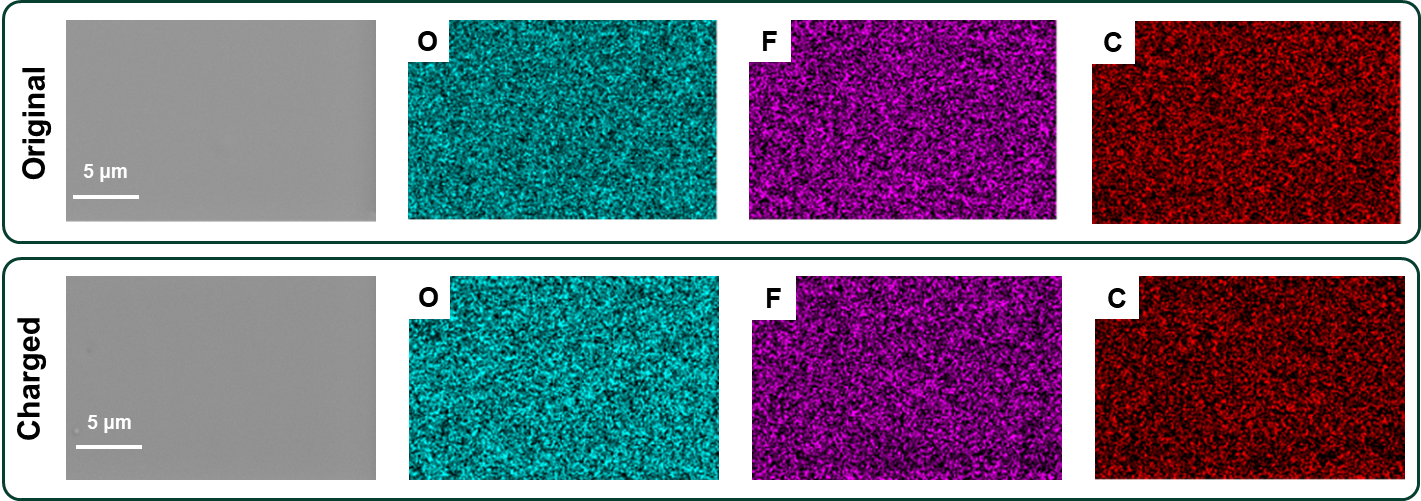


**Figure S6.** Morphological characterization and element mapping analysis of AF films before and after the EWCI.

**
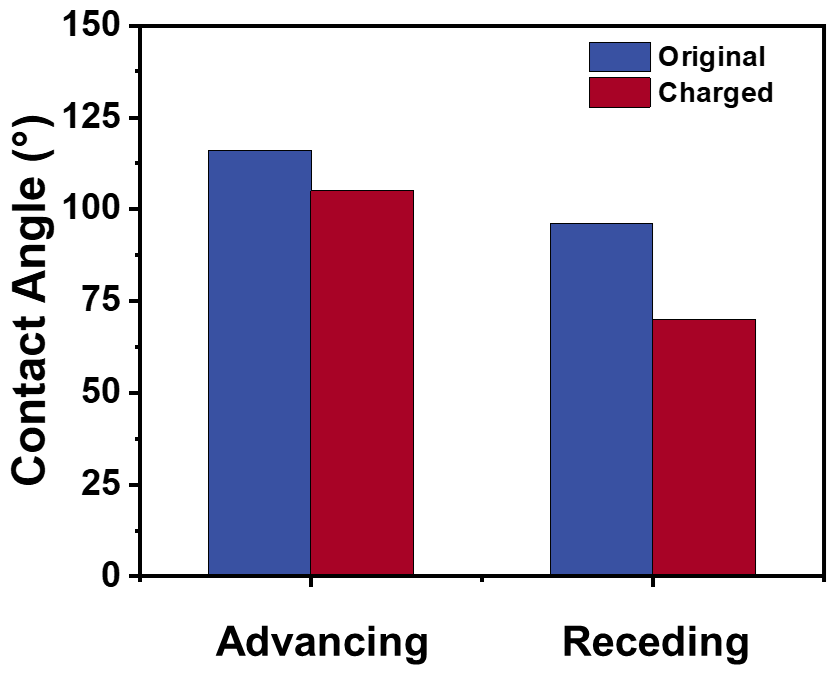
**

**Figure S7.** The forward angle and backward angle of Teflon AF1600 before and after EWCI process.

**Figure S8.** XPS spectra of AF films before and after EWCI process


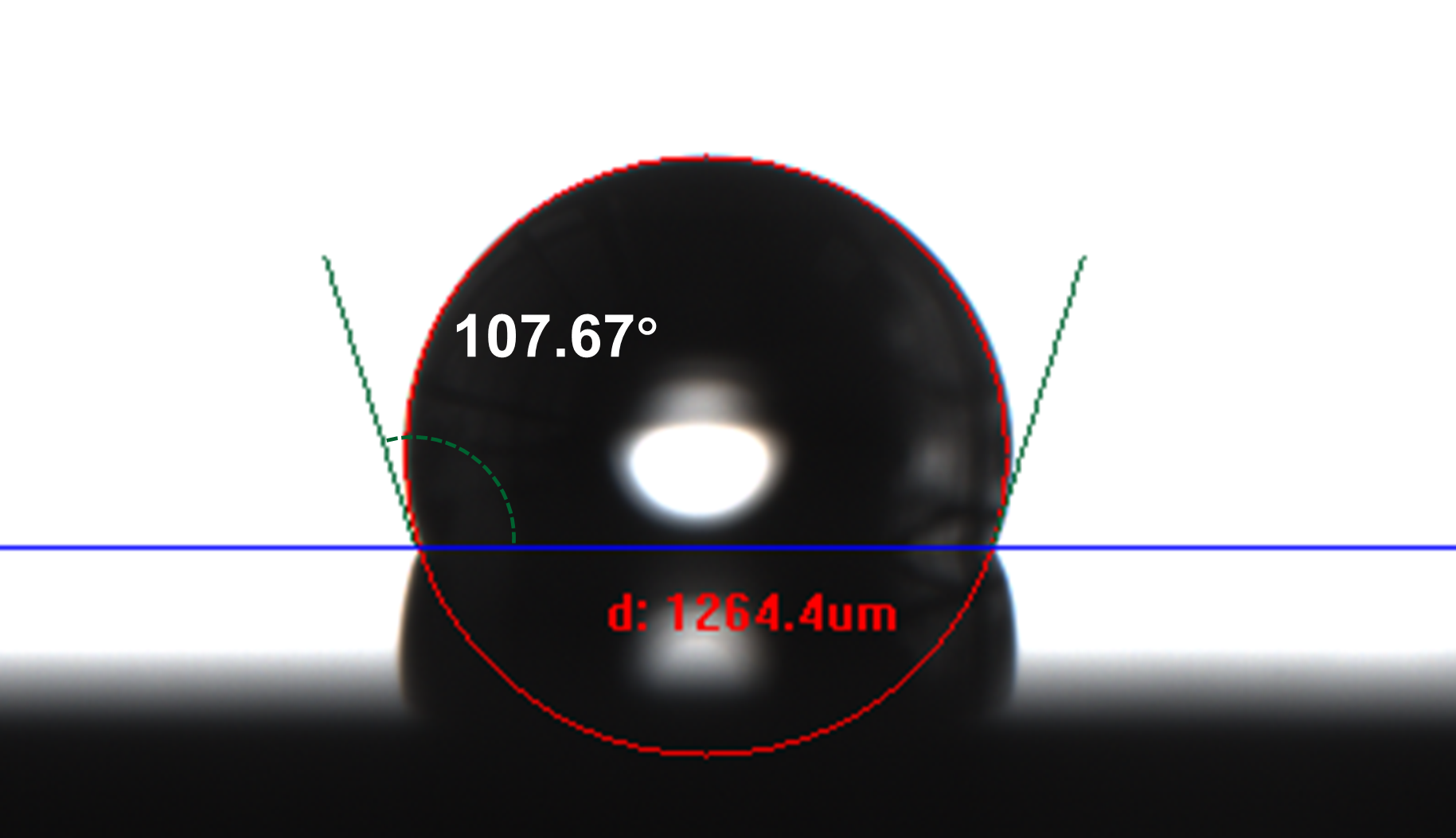


**Figure S9.** The contact angles of the water on the surface of FEP.

**
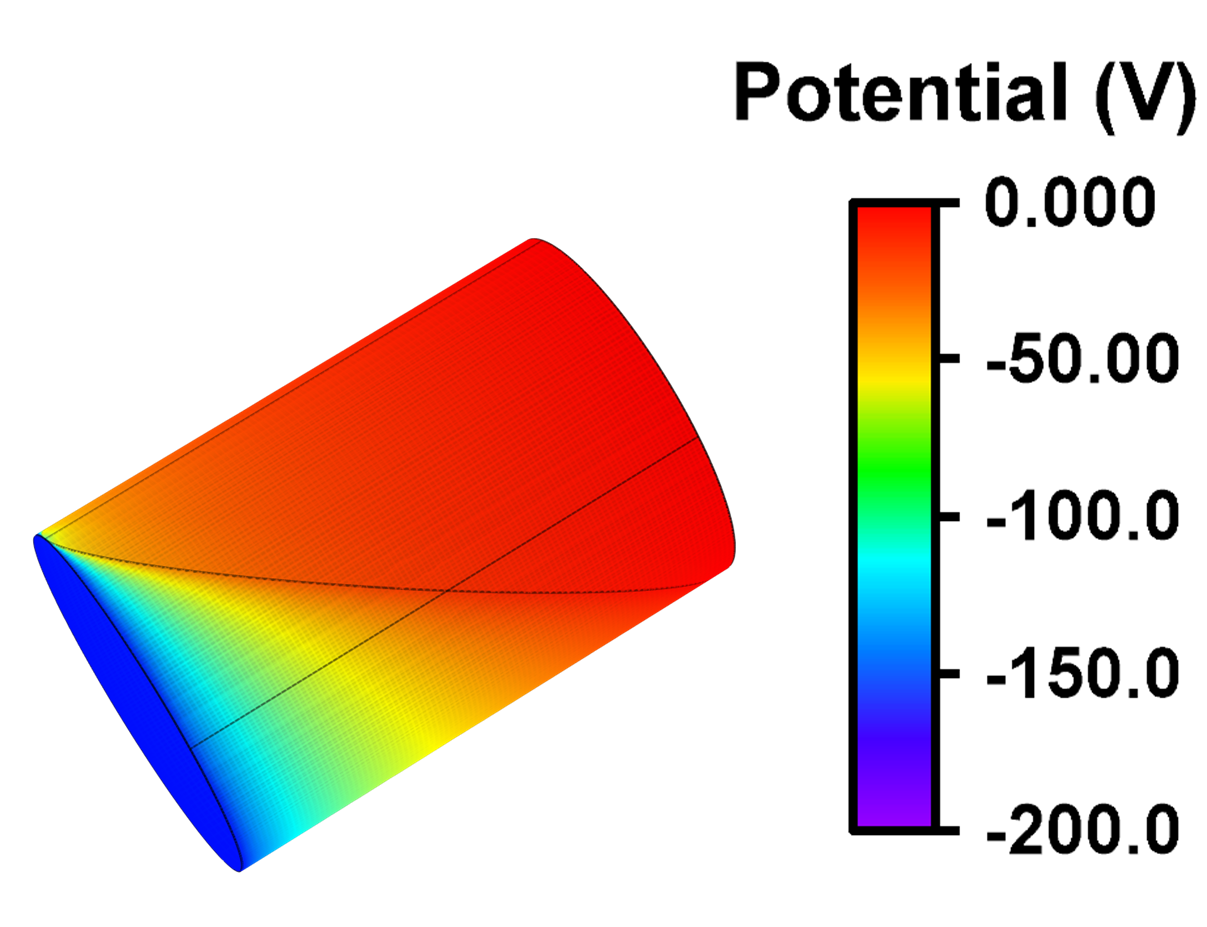
**

**Figure S10.** Potential distribution of EW-NG. The thickness of the SiO_2_ layer was 1000 nm.

The diameter of the conductive silicon was set to 30 mm. The distance between the two conductive silicon wafers was 30 mm. The thickness of the conductive silicon was 1000 nm. Water setting takes up half of the volume of the pipe (shape in the extreme position). The dielectric constants of air, water, and silica were 1, 81, and 3.9, respectively. The potential at the left conductive silicon was set to 0.


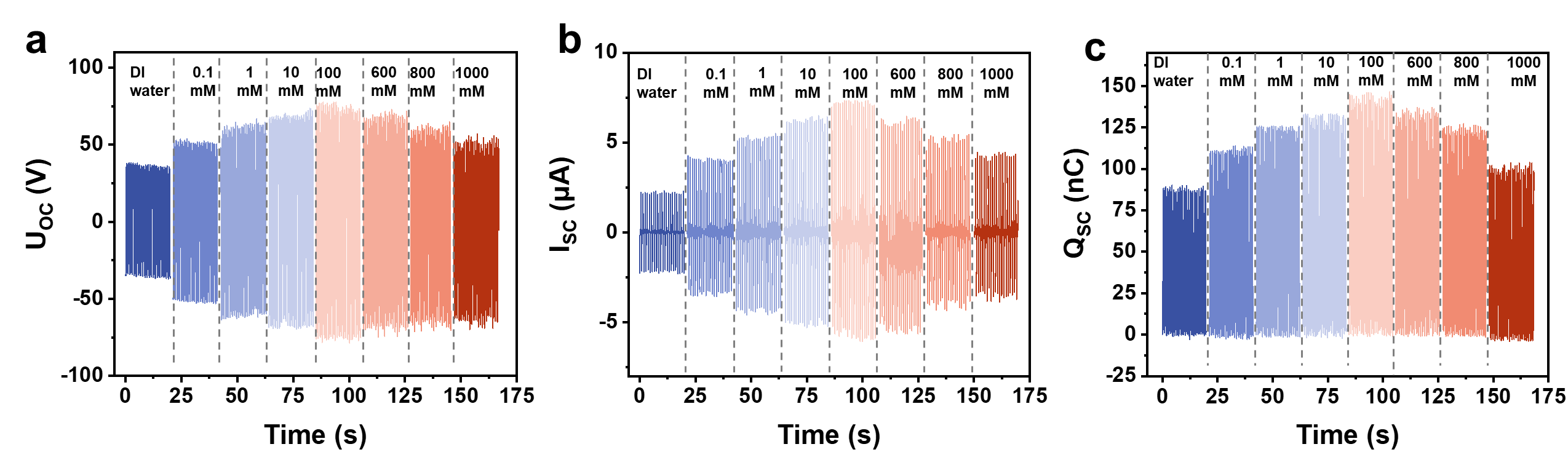


**Figure S11.** Electrical output of EW-NG with different concentrations of sodium chloride solution. (The length of the tube is 30 mm, the diameter is 20 mm, the volume of the solution is 30%, the swinging angle is 30, and the frequency is 2 Hz.)


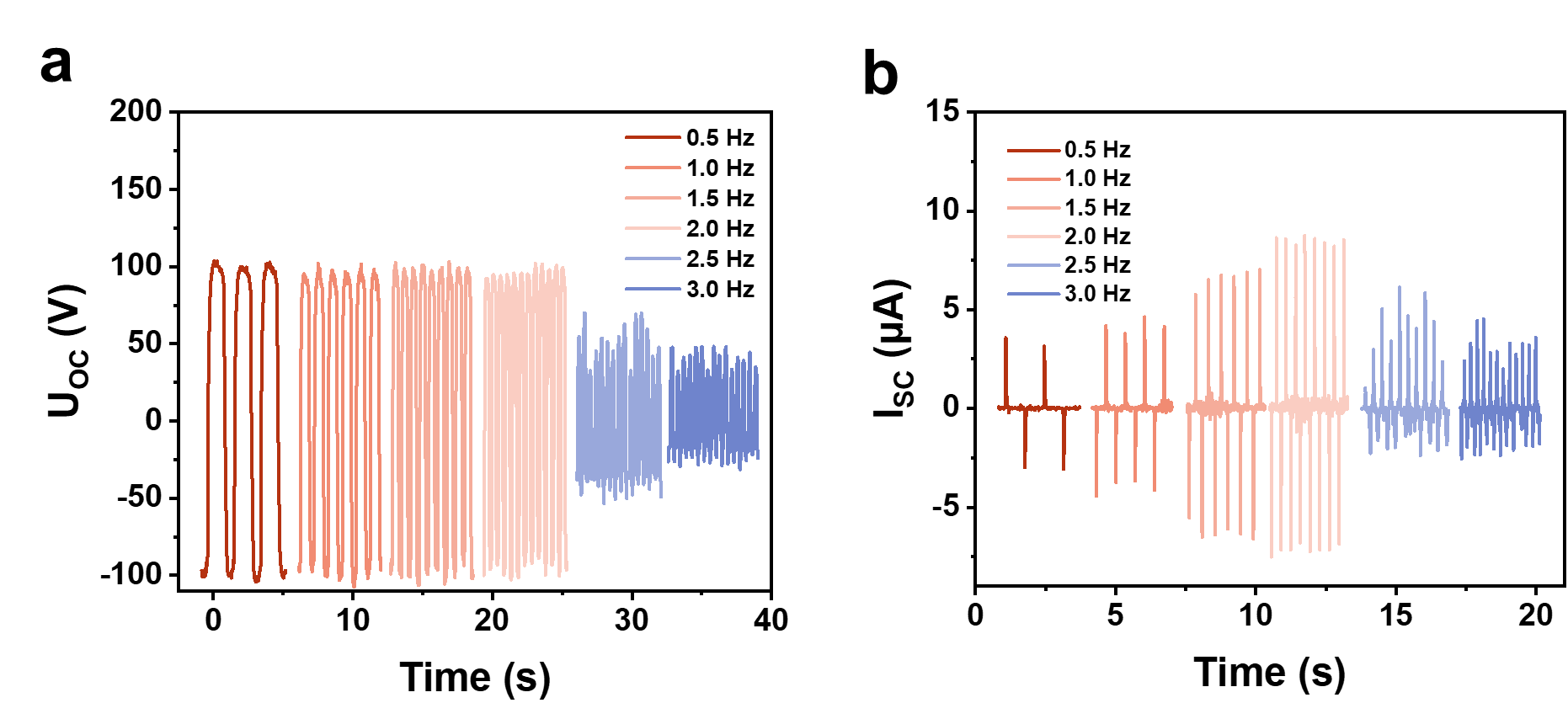


**Figure S12.** Influence of movement frequency on U_OC_ and I_SC_ of EW-NG in seesaw motion (30% volume fraction solution, 100 mM sodium chloride, swing angle 60°.)


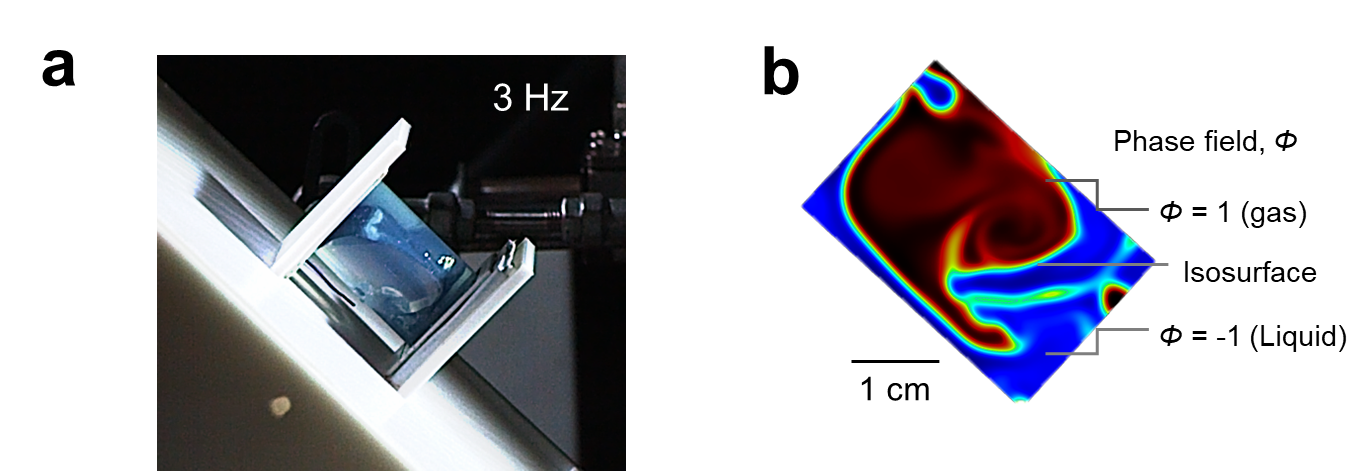


**Figure S13.** High-speed photo taken by EW-NG in seesaw fluid motion state at a) 3 Hz frequency. The corresponding frequency COMSOL simulation.

**Figure S14.** A larger EW-NG with a diameter of 34 mm and a length of 30 mm was prepared. The influence of frequency on the output was tested in seesaw mode with 40% solution volume fraction.


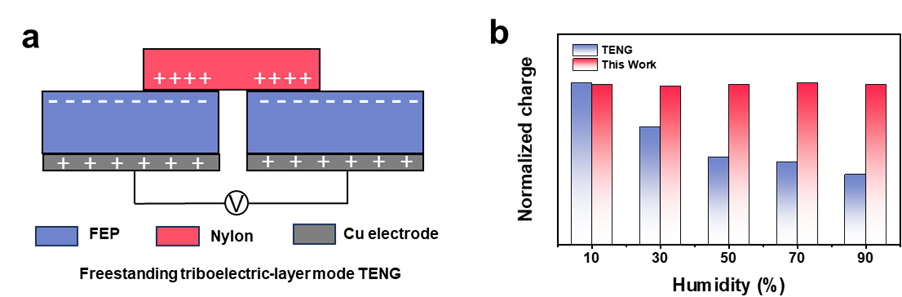


**Figure S15.** a. Structure and working principle of Freestanding triboelectric-layer mode TENG. b. Output of TENG and EW-NG at different humidities.

**Table S1.** Comparison of the volumetric charge output between this work and others

| No. | Volumetric Charge Density (mC/m^3^) | Authors |
| --- | --- | --- |
| 1 | 0.159 | X. Liang et al. ^[S1]^ |
| 2 | 1.4 | Z. Yuan et al. ^[S2]^ |
| 3 | 3 | S Jeon et al. ^[S3]^ |
| 4 | 4.3 | C. Xu et al. ^[S4]^ |
| 5 | 8.3 | Q. Zhou et al. ^[S5]^ |
| 6 | 9 | H. Zhang et al. ^[S6]^ |
| 7 | 9.5 | H. Wu et al. ^[S7]^ |
| This work | 19.1 | Y. Zhao et al. |

**References:**

[S1] X. Liang, T. Jiang, G. Liu, Y. Feng, C. Zhang, Z. L. Wang, *Energy Environ. Sci.* **2020**, *13*, 277.

[S2] Z. Yuan, C. Wang, J. Xi, X. Han, J. Li, S.-T. Han, W. Gao, C. Pan, *ACS Energy Lett.* **2021**, *6*, 2809.

[S3] S.-B. Jeon, D. Kim, M.-L. Seol, S.-J. Park, Y.-K. Choi, *Nano Energy* **2015**, *17*, 82.

[S4] C. Xu, Y. Liu, Y. Liu, Y. Zheng, Y. Feng, B. Wang, X. Kong, X. Zhang, D. Wang, *Appl. Mater. Today* **2020**, *20*, 100645.

[S5] Q. Zhou, B. Wang, A. Gao, W. Xu, K. Zhou, J. Pan, G. Meng, C. Pan, F. Xia, *Adv. Funct. Mater.* **2022**, *32*, 2209100.

[S6] H. Zhang, K. Wang, J. Li, J. Li, R. Zhang, Y. Zheng, *Matter* **2022**, *5*, 1466.

[S7] H. Wu, Z. Wang, Y. Zi, *Adv. Energy Mater.* **2021**, *11*, 2100038.
